# Non-diagnostic stereotactic intracranial biopsies: a 15-year institutional experience

**DOI:** 10.1007/s11060-026-05584-6

**Published:** 2026-04-21

**Authors:** Vratko Himic, Sebastian Vargas-George, Maxon V. Knott, Vaidya Govindarajan, Seth S. Tigchelaar, Tyler M. Cardinal, Adham M. Khalafallah, Victor M. Lu, Hatun Mine Sahin, Arnold Lang, Zachary C. Gersey, Daniel M. Aaronson, Ricardo J. Komotar, Ashish H. Shah, Michael E. Ivan

**Affiliations:** 1 Department of Neurological Surgery, University of Miami Miller School of Medicine, Miami, FL USA; 2 Department of Neurosurgery, Loma Linda University Medical Center, Loma Linda, CA USA; 3 Division of Neurological Surgery, Saint Louis University School of Medicine, St. Louis, MO USA

**Keywords:** Biopsy, Brain, Stereotactic, Non-diagnostic, Robot-assisted

## Abstract

**Purpose:**

Stereotactic brain biopsies are not without risk, and non-diagnostic biopsies can delay the instigation of appropriate treatment. Institutions rarely report their negative (non-diagnostic) results; this reporting is essential for quality and improvement in neurosurgical departments.

**Methods:**

We report a retrospective single-center decade-long cohort of non-diagnostic stereotactic biopsies. We report the indications for biopsy, histopathological findings, rates of re-biopsy, lesion characteristics and biopsy success metrics such as depth-do-diameter, biopsy accessibility metrics and true (TN) and false (FN) negative rates.

**Results:**

Of 387 biopsies, 23 were non-diagnostic (5.94%). Among 196 robot-assisted procedures, the non-diagnostic rate was 5.05%, compared to 6.88% across 189 non-robot-assisted biopsies (*p* = 0.52). The anticipated diagnosis was tumor/metastasis in 65% of cases. 26% of patients were asymptomatic prior to biopsy. The target was subcortical in 61% of cases, with median volume 8.36cm^3^ and lesion depth 65.05 mm. Deeper lesions were larger (*r* = 0.47, 95%CI:0.02–0.76, *p* = 0.036). Biopsy showed indeterminate mild gliosis in 60.9%, normal parenchyma in 34.8% and hemorrhage in one case. Two patients underwent re-biopsy. Post-biopsy, 52% were treated medically, 26% remained under observation, the remainder were palliated or underwent surgery. On review of prior and future clinical/radiological/histopathological data, 7 (30.4%) were TN, 14 (60.8%) were FN. No TN biopsies occurred at a lesion depth of less than 50 mm.

**Conclusions:**

The optimal method and equipment for stereotactic biopsies remains an area of personal and institutional preference. Internal review of these metrics can identify areas for improvement of service delivery in a procedure that is central to the diagnosis and treatment of patients with brain lesions.

## Introduction

Stereotactic biopsy enables a minimally invasive diagnostic opportunity characterized by shorter operative time, smaller incisions and rapid access to the target lesion. It offers significant diagnostic advantages in small or radiologically indeterminate lesions located within eloquent or functionally critical brain areas. Despite its clinical utility, the safe acquisition of an adequate quantity of diagnostic tissue with a biopsy needle remains a technical challenge [1], and its success is a combination of skill, technique, meticulous planning and to some extent, some surgical *gestalt*.

Recent technological advancements have aimed to reduce operator variability and increase biopsy accuracy in stereotactic procedures. However, the difficulty of obtaining non-diagnostic tissue remains a challenge. Despite the relevance of non-diagnostic biopsies, few institutions have published dedicated reviews of these cases, with many series focusing on technical success and diagnostic accuracy, while underreporting the burden and implications of failure [2–8]. Furthermore, current guidelines emphasize procedural technique rather than guidance regarding post-biopsy management in cases of inconclusive histopathology [9]. This lack of literature and guidance not only affects clinical decision-making but also hampers the ability to improve protocols and reduce avoidable diagnostic delays.

The clinical trajectory of patients following a non-diagnostic biopsy remains sparse in the literature, suggesting re-biopsy is often successful, with conversion rates above 75% reported [7, 10]. However, outcomes for patients managed without a definitive diagnosis remain less studied, and institutional variability in follow-up strategies complicates the development of universal guidelines.

In this retrospective single-center study, we report a cohort of non-diagnostic stereotactic intracranial biopsies across a 15-year period. We systematically analyze clinical indications, lesion characteristics, procedural methods and post-biopsy clinical outcomes. In addition, we carry out a sub-analysis of those non-diagnostic biopsies that were truly negative (TN) and those that were falsely negative (FN).

The reporting of negative (non-diagnostic or undesirable) results is relatively rare in the literature, and we do so in an effort to improve transparency, improve our service delivery, and continue inter-specialty dialogue on how to manage non-diagnostic stereotactic brain biopsies.

## Methods

### Clinical details, radiological data, and definitions

We performed a single-center retrospective case series including patients who underwent a stereotactic intracranial biopsy for intracranial lesions between 2011 and 2024. Non-diagnostic cases were defined as those with failure to provide any lesion-specific pathology. For the overall biopsy cohort of all patients, available variables included biopsy method (ROSA robotic vs. non-robotic), lesion laterality, general lobar location, grouped anatomical location (cortical, subcortical, or cerebellar), and year of surgery.

For the non-diagnostic subgroup, additional clinical and radiographic variables were available, including presenting symptoms, postoperative follow-up, and lesion characteristics such as maximal diameter, lesion volume, biopsy depth, and imaging visibility (contrast-enhancing vs. non-enhancing lesions). Further histopathological and radiological data collected included lesion biopsy depth, volume, and largest diameter. To characterize technical difficulty of lesion targeting, a depth-to-diameter ratio was calculated by dividing lesion depth from the cortical surface by maximal lesion diameter. Higher depth-to-diameter ratios indicate smaller and deeper lesions and were used as a surrogate marker of stereotactic sampling difficulty. Re-biopsy status (and its relative success/failure) and final diagnosis were also documented. Last follow-up dates, subsequent treatment and pathology notes for non-diagnostic cases, as well as patients’ clinical course including survival status were also reviewed.

Of those patients with a non-diagnostic biopsy, true (TN) or false (FN) negative status was determined. A FN biopsy was defined as that which had previously or subsequently had a diagnostic biopsy at another timepoint, or that for which the subsequent post-biopsy clinical course and symptomatology was clearly linked to the underlying CNS lesion that was non-diagnostic and for which the patient received treatment. Any biopsy not meeting these criteria and for which no further management was needed was defined as a TN biopsy. This study was approved by the Institutional Review Board at the University of Miami (IRB#20160437). Given the retrospective nature of this chart review, this data collection was approved by our IRB without the need for explicit patient consent.

### Biopsy procedure

All procedures were performed under general anesthesia. Each patient was positioned on the operating table according to the planned trajectory, with the head secured in the corresponding stereotactic system. Three-dimensional contrast-enhanced MRI scans were obtained one day prior to surgery, and uploaded into the stereotactic system, which was used to assist trajectory and entry point planning. Fiducial markers were applied based on the surgeon’s preference. Following coordinate registration, trajectory confirmation was performed using an intraoperative O-arm scan. The stereotactic system that was used (either the ROSA robotic system or a non-ROSA navigation system (ROSA ONE^®^ Brain, Zimmer Biomet, Warsaw, Indiana, USA)) was recorded. In each case, several biopsy cores were obtained using the biopsy needle compatible with the respective system. Sampling sites included several points along the planned trajectory. All patients were monitored either on the inpatient floor or the neurosurgical intensive care unit overnight following the procedure. Postoperative MRI was acquired the next day for every patient. In the absence of postoperative complications, patients were discharged on postoperative day 1.

### Statistical analysis

Continuous variables were expressed as mean ± standard deviation (SD) or median with interquartile range (IQR) depending on normality of data distribution. Diagnostic and nondiagnostic biopsies were compared using Fisher’s exact test due to small, expected cell counts in the nondiagnostic group. A secondary descriptive analysis was performed for the nondiagnostic biopsy cohort; these variables were summarized using descriptive statistics. A tertiary final analysis involved descriptive statistics between TN and FN biopsies within the non-diagnostic sub-cohort. Relationships between continuous variables within the nondiagnostic cohort were explored using correlation analysis with Spearman’s rank correlation coefficient. Data distributions were visualized using histograms, scatter plots, and box-and-whisker plots as appropriate. Statistical analyses were performed using GraphPad Prism (GraphPad Software, San Diego, CA). All statistical tests were two-sided, and a p-value < 0.05 was considered statistically significant.

## Results

**Fig. 1:**
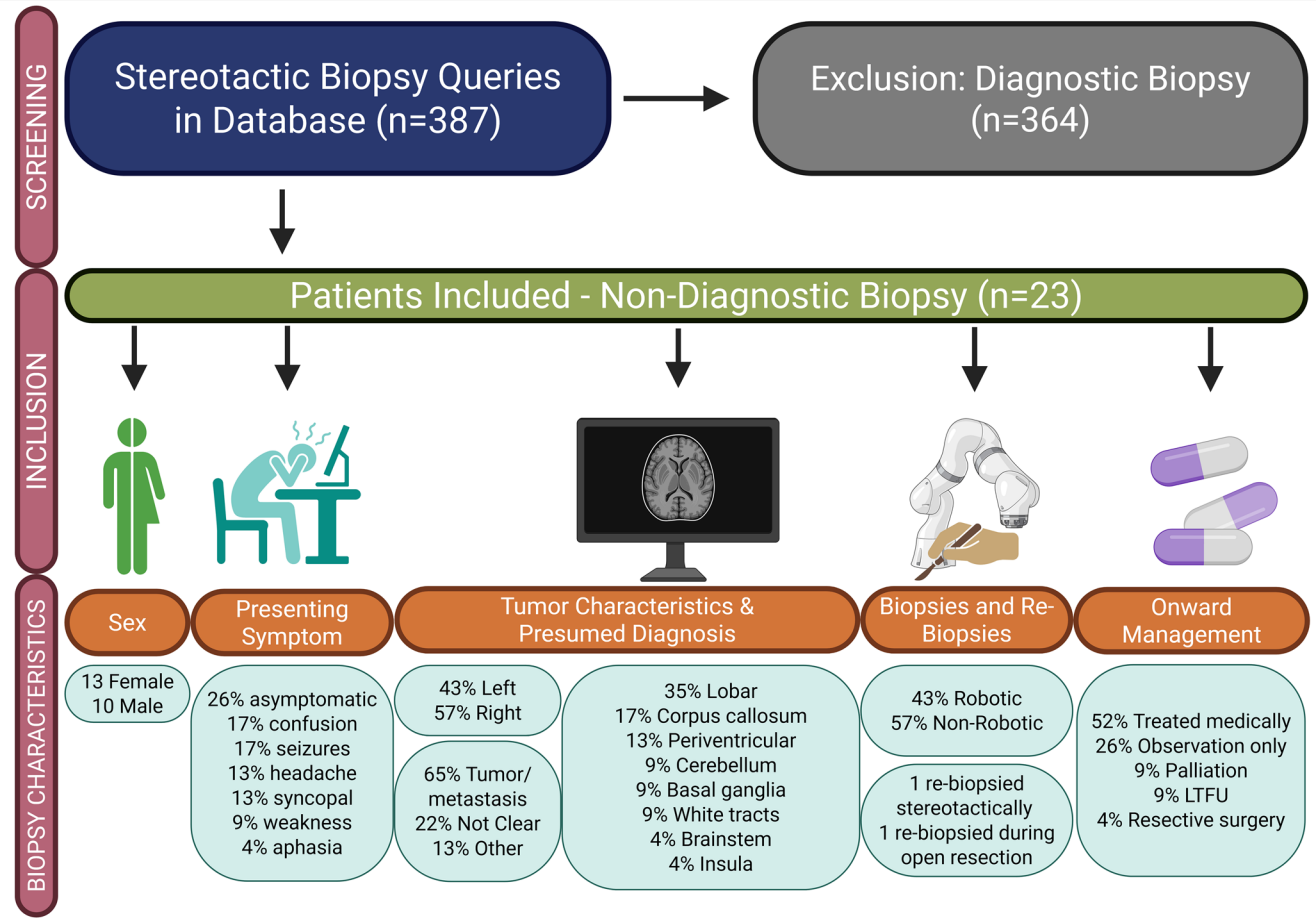
Patient selection and descriptive summary of non-diagnostic biopsy cases: presentation, lesion characteristics and onward management

Out of 387 patients with stereotactic biopsy, 23 had a non-diagnostic biopsy (Fig. 1), accounting for a diagnostic yield of 94.1% and an overall non-diagnostic rate of 5.94%. Within the group of non-diagnostic biopsies, 57% (*n* = 13) were female and 43% (*n* = 10) were male (Table 1). Among symptomatic patients, the most frequent manifestations were cognitive decline, seizure, and headache, each accounting for 13–17% of all cases. Nevertheless, 26% of patients were asymptomatic at presentation. In 65% of cases the presumed pre-biopsy diagnosis was either a tumor or metastatic (neoplastic) lesion. In 22% of cases there was no convincing anticipatory diagnosis before biopsy. The remaining three cases (13%) were postulated to be due to granulomatous disease, multiple sclerosis (MS), and acute disseminated encephalomyelitis (ADEM).

**Table 1:** Breakdown of total diagnostic and negative biopsy counts across the study period, including non-diagnostic biopsy percentage breakdown.

	Total	364	23	387	Non-diagnostic rate	p-value
		Diagnostic	Non- diagnostic	Total		
Laterality	Left	161	10	171	5.85%	> 0.999
	Right	203	13	216	6.02%	
Method	Non-Robotic	178	13	191	6.81%	0.55
	Robot-assisted	186	10	196	5.10%	
Year	2011	3	0	3	0.00%	*r* = 0.207 *p* = 0.474
	2012	7	0	7	0.00%	
	2013	18	0	18	0.00%	
	2014	19	1	20	5.00%	
	2015	18	0	18	0.00%	
	2016	26	2	28	7.14%	
	2017	30	7	37	18.92%	
	2018	23	2	25	8.00%	
	2019*	22	3	25	12.00%	
	2020	37	3	40	7.50%	
	2021	45	3	48	6.25%	
	2022	54	0	54	0.00%	
	2023	43	1	44	2.27%	
	2024	23	1	24	4.17%	

Statistical analysis represents Fisher’s exact test or Spearman’s rank correlation coefficient. *Robot-assisted biopsies were introduced in 2019

### Robotic vs. non-robotic biopsy

**Fig. 2:**
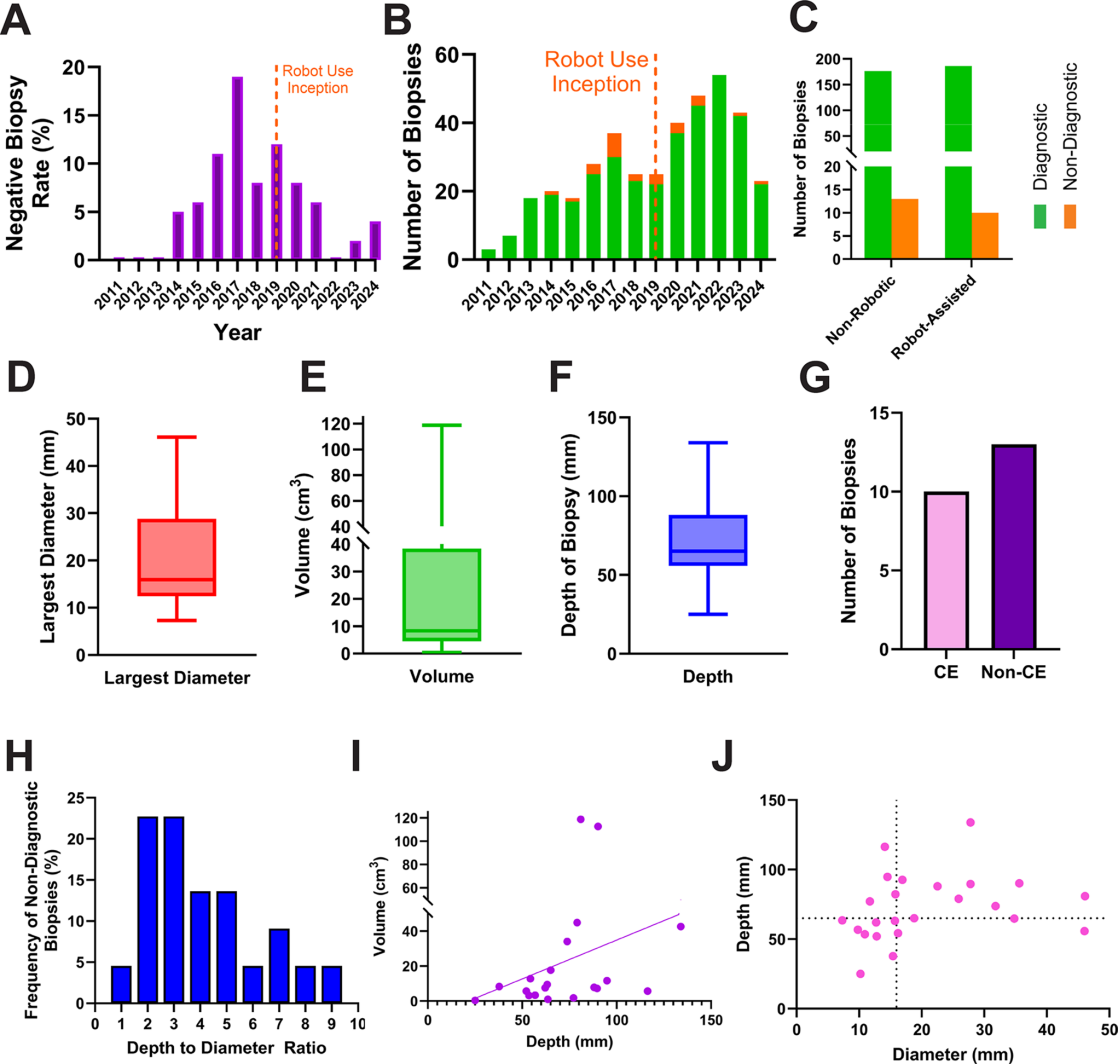
Diagnostic and non-diagnostic biopsies over time.** A** – The rate of non-diagnostic biopsy stratified by year from 2011 to 2024. Robot-assisted biopsy began in 2019. **B** – The raw number of biopsies that were diagnostic and non-diagnostic over the same period. **C** – Diagnostic and non-diagnostic biopsy breakdown based on Robot use or not (the difference was not statistically significant). **D-E** – The distribution of the largest diameter (**D**), volume (**E**), and depth (**F**), of non-diagnostic biopsied lesions. **G** – The number of non-diagnostic biopsies broken down by those that enahced (CE) and did not enhance (Non-CE) on T1 MR imaging. **H** – Histogram of non-diagnostic biopsies based on the depth-to-diameter. **I** – Scatter plot of depth and volume of each lesion, with a significant Spearman correlation ration indicating deeper lesions tended to be larger in terms of volume. **J** – Diameter versus depth biopsy accesibility plot, with four quadrants divided by the median diameter and depth of the non-diagnostic biopsy cohort. No correlation that would suggest that a particular combination (e.g. deeper and smaller lesions in the upper left quadrant) would have greater rates of non-diagnosis

Both robotic (ROSA-assisted) and conventional non-robotic stereotactic platforms were employed over the 15-year study period (Fig. 2a). The ROSA robotic system was used in 43% (*n* = 10) of non-diagnostic biopsies. There was a clear delineation of these cases in the study period, with all non-diagnostic biopsies between 2011 and 2018 being non-ROSA, whilst from 2019 onwards all, bar one, negative biopsies were using ROSA, mirroring changes in our practice over time (Fig. 2b). Among 196 ROSA-assisted procedures, the non-diagnostic rate was 5.05% versus 6.88% across 189 non-ROSA biopsies (*p* = 0.52) (Fig. 2c).

### Pre-operative radiological findings

The target lesion was located on the left side in 43% (*n* = 10) of cases, on the right side in 57% (*n* = 13). In terms of anatomic distribution, 61% (*n* = 14) were subcortical, while 39% (*n* = 9) were lobar (frontal, temporal, occipital) or cerebellar. The median (IQR) values for largest diameter, volume and lesion volume in the cohort were 15.95 mm (IQR:12.45–28.80), 8.36cm^3^ (IQR:4.54–38.40), and 65.05 mm (IQR:55.75–88.01) respectively (Fig. 2d-f). The lesion displayed contrast-enhancement in 10 out of 13 cases (Fig. 2g). On depth-to-diameter ratio analysis, most biopsy ratios clustered between 2 and 5, and tapered off towards higher numbers, not consistent with the hypothesis that smaller deeper lesions would necessarily increase negative biopsy difficulty (allowing for small counts) (Fig. 2h and j). Deeper lesions tended to be larger in volume (*r* = 0.47, 95%CI:0.02–0.76, *p* = 0.036) (Fig. 2i).

### True versus false negative sub-analysis

**Fig. 3:**
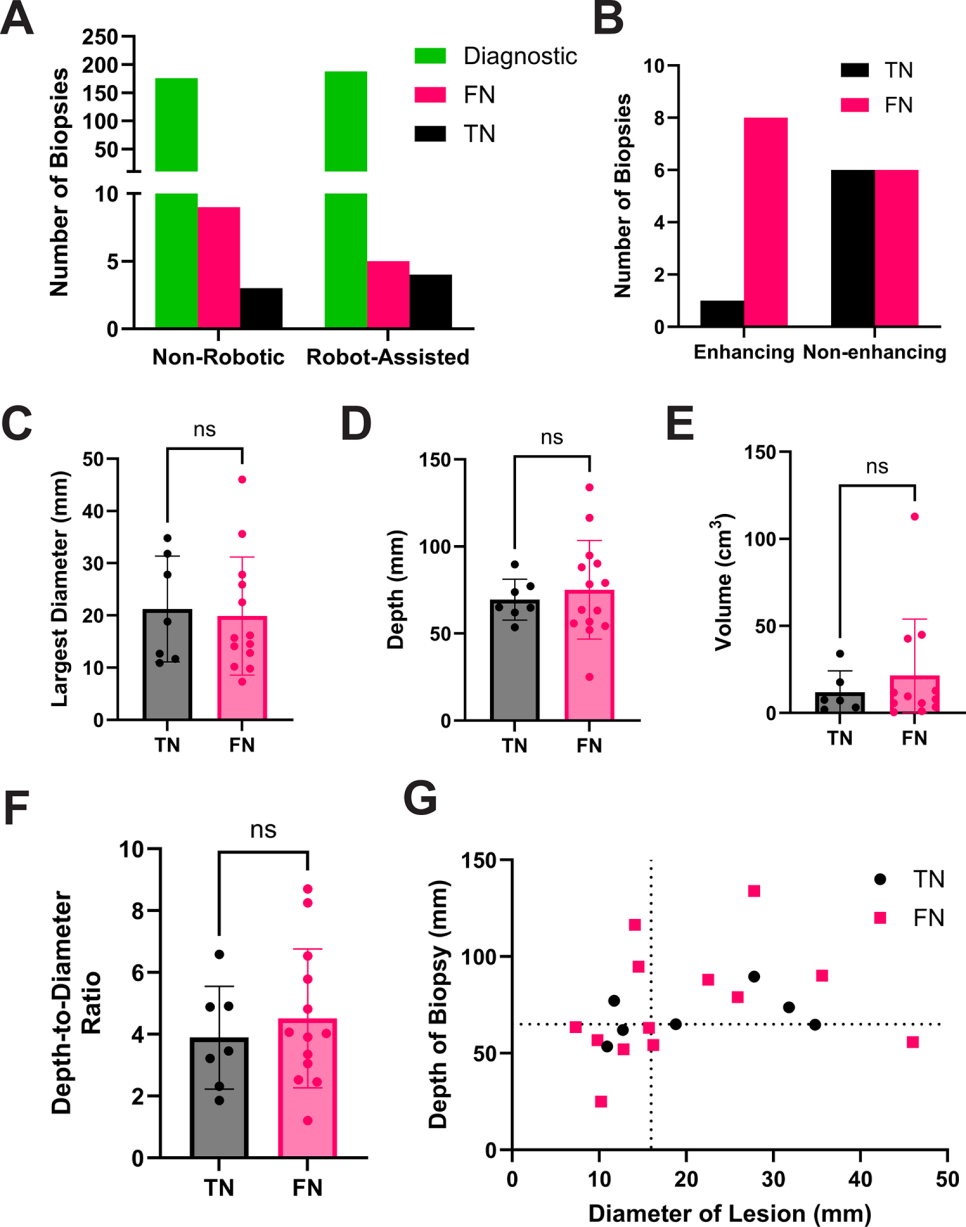
Non-diagnostic biopsies further broken down into true negatives and false negatives.** A** – Breakdown of the number of biopsies that were diagnostic, falsely negative and truly negative across the study period by robot use (these changes were not statistically significant). **B** – Breakdown of truly and falsely negative non-diagnostic biopsies by contrast enhancement on T1 MR imaging. **C-F** – Comparison of the mean and standard deviation of the largest diameter (**C**), depth (**D**), volume (**E**), and depth-to-diameter ratio (**F**), of non-diagnostic biopsied lesions by true (TN) or false (FN) negative status. **G** – Diameter versus depth biopsy accesibility plot, with four quadrants divided by the median diameter and depth of the non-diagnostic biopsy cohort. No correlation that would suggest that a particular combination (e.g. deeper and smaller lesions in the upper left quadrant) would have greater rates of non-diagnosis

Of the 23 patients with a non-diagnostic biopsy, 7 (30.4%) were TN, 14 (60.8%) were FN and 2 (8.70%) were lost-to-follow-up as it was not possible to determine through chart review whether the index biopsy was true or falsely negative (Fig. 3a) (Table 2). Taken together, this represents a total FN rate of 3.62% out of all biopsies. Regarding anticipated diagnosis broken down by TN and FN status, we report the following breakdown. TN patients had a 71% (*n* = 5/7) presumed diagnosis of tumor/metastasis with the remaining two being unclear and ADEM. FN patients had a 64% (*n* = 9/14) presumed tumor/metastatic diagnosis, 21% (= 3/14) unclear and the two remaining cases (*n* = 2/14) were presumed to be MS or a granulomatous process. There was no difference in the presence of contrast enhancement between TN and FN lesions (*p* = 0.159) (Fig. 3b). Likewise, lesions in the FN and TN cohort were similar in their largest diameter, depth, volume and depth-to-diameter ratio (Fig. 3c-f). Of note, no TN biopsies occurred at a lesion depth of less than 50 mm (Fig. 3g) and only two non- diagnostic biopsies occurred at depths of less than 50 mm (Fig. 2j).

**Table 2:** Breakdown of true negative and false negative counts in the non-diagnostic biopsy cohort

	Total	7	14	*p*-value
		TN	FN	
Age (median)		56.78	55.4	0.322
Method	Non-Robotic	3	9	0.554
	Robot-assisted	4	5	
Lesion	Depth (mm)	65.05	70.9	0.799
	Diameter (mm)	18.8	15.7	0.772
	Volume (cm^3^)	7.41	8.71	0.750
	DDR	3.46	4.03	0.699
	Enhancing	1	8	0.159
	Non-enhancing	6	5	
Location	Superficial	5	5	0.141
	Deep	1	9	

7 were TN and 14 were FN, with 2 lost to follow up and therefore unable to determine true/false status. Statistical analysis represents Fisher’s exact test. TN – true negative; FN – false negative; DDR: depth-to-diameter ratio

### Histopathological findings during biopsy

We reviewed the pathological reports for each negative biopsy to identify whether we could identify a reason or description for the negative biopsy. Seven cases had intraoperative frozen biopsies taken, which were likewise non-diagnostic. Some form of non-specific and non-contributory mild gliosis was reported in 14 (60.9%) cases (although this remained non-diagnostic given the global picture). 8 cases (34.8%) had no mention of any abnormality apart from normal tissue and 1 case had non-diagnostic hemorrhagic material.

### Patient outcomes post-non-diagnostic biopsy

Alongside describing the presenting symptoms and clinical diagnosis before biopsy, we also wanted to assess the onward trajectory of patients who have a negative biopsy. The median follow-up duration in those with follow-up was 36 m (IQR:5.9–53.6 m), with two patients lost to follow up shortly after biopsy. No complications arose as a consequence of the biospies in our non-diagnostic cohort. Overall, 52% (*n* = 12) were treated medically and 26% were under observation only (Fig. 1). The remaining patients were palliated (*n* = 2, 8%), underwent surgery (*n* = 1, 4%) or were LTFU (*n* = 2, 9%).

Of those patients that underwent medical treatment, two were from the TN cohort and 10 were from the FN cohort. The two TN patients underwent monoclonal antibody and steroid treatment for suspected paraneoplastic disease. This diagnosis was solidified on subsequent repeat imaging and clinical evaluation; accordingly, the biopsied lesion was determined not to be consistent with the rest of the disease process. Of the 10 FN patients who were treated medically, this involved for disease ranging from NF1, non-Langerhans cell histiocytosis, glioma, and infarct, through to vasculitis, primary angiitis, Parkinson’s disease and MS. Of those patients who were only under observation (*n* = 6/23), most (*n* = 5) were TN and one (*n* = 1) was FN. The one FN patient who underwent no further treatment had a prior diagnosis and repeat resection of pilocytic astrocytoma, and as they were asymptomatic, an observation recommendation was made.

Both patients who were palliated progressed rapidly after their non-diagnostic biopsy. One was known to have a glioma from a prior biopsy and the other was presumed to have a metastasis due to strong clinical suspicion and radiological progression. Both experienced significant neurological dysfunction with aphasia and confusion. Neurological outcomes worsened in 3 of the 25 patients (12%), 2 developed worsening weakness and 1 developed both weakness and visual deficits. All three of the patients with worsening neurological status were in the FN cohort. Re-biopsy was performed in 2 (9%) of non-diagnostic biopsy patients. One was taken stereotactically and remained non-diagnostic, whereas the second was taken during an open resection and was diagnostic for lymphoma. At last available follow-up, 24% (*n* = 6) of patients had died.

## Discussion

We describe an institutional retrospective descriptive review of our department’s 15-year experience with stereotactic biopsies. Our aim was to describe the peri-biopsy clinical and etiological factors, as well as the analysis of patients who might have developed adverse neurological dysfunction because of non-diagnostic biopsy. We report an overall diagnostic yield of 94.06%, a nondiagnostic rate of 5.94%, alongside a false negative fraction of 60.87% in our 23-patient cohort. Seven other studies explicitly reported non-diagnostic biopsy rates, ranging 2.6%-13.8% (Table 3) [2–7]. We strove to share our institutional experience with the challenging clinical conundrum of what to do with a non-diagnostic biopsy.

### Patient presentation and indications

26% of non-diagnosed patients presented asymptomatically, followed by headache, confusion, and seizures. Clinical manifestations were largely non-specific and did not correlate with the final diagnosis, as many lesions were discovered incidentally, consistent with previous reports [11, 12]. Stereotactic biopsies are typically performed for small and/or radiologically indeterminate lesions, and it is therefore expected that many patients present asymptomatically; this is factored into decision making when choosing *not* to perform a resection or laser ablation of the lesion. In our cohort, the primary indication for stereotactic biopsy was suspicion of neoplasm or metastasis (65%). Histopathologically, non-neoplastic processes may yield nonspecific or reactive changes, which can be difficult to interpret and may not provide a definitive diagnosis without correlation to clinical and laboratory data [11, 13, 14]. Non-neoplastic lesions are associated with higher rates of non-diagnostic results in stereotactic intracranial biopsy; in one series, the diagnostic yield for non-neoplastic lesions was 73.3%, significantly lower than the 91.2% yield for tumors [7]. Non-neoplastic lesions often lack the well-demarcated, contrast-enhancing features typical of neoplasms, making accurate targeting more challenging [7, 11, 15].

**Table 3:** Literature Review of studies that explicitly report non-diagnostic biopsy rates

First Author (Year)	Study Design & Cohort	Non-diagnostic Biopsy Rate	Key Findings on Negative Biopsies	Ref.
Hall (1998)	Retrospective, 134 biopsies	4%	Failures due to lesion location (ventricular), inaccurate targeting, inability to penetrate tumor	[2]
Dammers (2008)	Retrospective, 391 biopsies	10.6%	Left-sided lesions less likely negative; cerebellar lesions high risk for negative histology	[3]
Dammers (2010)	Retrospective, 164 biopsies	11% (to 1.8% with frozen)	On-demand intra-op frozen section reduced negative rate from 11% to 1.8%	[4]
Livermore (2014)	Retrospective, 351 biopsies	5.1%	Higher negative rate in deep/cerebellar lesions; intra-op smear reduced negative rate to 3.7%	[5]
Mathon (2019)	Retrospective, 145 biopsies	2.6% (to 0% with smear)	Intra-op smear reduced negative rate from 2.6% (historical) to 0%	[6]
Marakgos (2020)	Retrospective, 198 biopsies	5.6%	Lesion diameter was the only independent predictor of diagnostic yield, but the overall diagnostic yield remained high (94.4%) even with a wide range of lesion sizes	[16]
Pasternak (2021)	Retrospective, 311 biopsies	13.8%	Small size, non-enhancing lesions, sepsis, hemato-oncologic disease increase risk; re-biopsy diagnostic in 75%	[7]
This Cohort (2026)	Retrospective, 387 biopsies	5.94%	Our study found false negative fraction of 60.87%, with only 7 of 23 non-diagnostic biopsies being truly negative.	

Range from 2.6–13.8%, with our cohort having a non-diagnostic rate of 5.94%

### Lesion characteristics

61% of non-diagnostic biopsies involved deep-seated or subcortical targets. A depth-to-diameter ratio was calculated as a surrogate of lesion accessibility, relating target depth to lesion size. Most lesions demonstrated ratios between 2 and 5, with relatively few extreme values (Fig. 2h). On inspection of the biopsy accessibility plots, nondiagnostic biopsies did not cluster in the region corresponding to small, deep lesions with higher ratios (Fig. 2j). Although limited by the small cohort size, this finding does not support the assumption that geometric targeting difficulty alone accounts for nondiagnostic biopsy. Notably, deeper lesions in this cohort also tended to be larger in volume (Fig. 2i), suggesting that increased lesion size may partially offset the technical challenges associated with longer trajectories. It is important to note that whilst we did not identify a significant association between lesion depth or depth-to-diameter ratio and non-diagnostic outcomes, this should be interpreted cautiously, as deeper lesions impose practical constraints on trajectory planning and sampling that are not fully captured by simplified measures such as these, including the compromises that must be made along the biopsy trajectory. With increasing depth, the likelihood of having to calculate and plan to avoid each neurovascular structure increases. In addition, with each degree of marginal error that is inherent to all techniques (including using the robot), each degree of inherent target uncertainty magnifies as the catheter goes deeper.

Further stratification of nondiagnostic biopsies demonstrated that the majority represented FN rather than TN. When comparing TN and FN lesions, there were no clear differences in imaging characteristics, including contrast enhancement, lesion size, depth, volume, or depth-to-diameter ratio (Fig. 3c-f). These findings suggest that commonly cited factors related to lesion accessibility (smaller and/or deeper targets) were not strongly associated with FN sampling in this series. Interestingly, no TN biopsies occurred in lesions shallower than 50 mm (Fig. 3g). Taken together, these observations suggest that geometric targeting constraints alone are unlikely to explain most nondiagnostic outcomes in this cohort.

Several series have suggested that lesion volume or diameter is a significant predictor of diagnostic yield, with smaller lesions more likely to result in nondiagnostic biopsies. However, the magnitude of this effect can vary depending on other factors such as lesion location, imaging characteristics. One study reported that lesion diameter was the only independent predictor of diagnostic yield [16]. Small lesion size can also be a predictor of nondiagnostic biopsy [7]. The small size and the often diffuse or infiltrative nature of these deep lesions can increase the likelihood of non-diagnostic or inconclusive specimens [17, 18]. Even with advanced imaging guidance, the need to avoid vasculature and functional areas may constrain the biopsy trajectory, further limiting tissue acquisition.

The risk of neurological morbidity increases with attempts to access these deeper areas, often driving surgeons to pursue minimally invasive or stereotactic approaches that tend to limit the volume and quality of tissue obtained, reducing diagnostic yield [17–19]. These are some of the reasons for exercising extreme caution when considering re-biopsy of a lesion. Recent innovations have been introduced to reduce the challenges inherent to these locations and to improve access and safety, but the risk naturally remains [19, 20].

### The biopsy process

Several procedural and temporal factors warrant further consideration when interpreting the findings of this cohort. Firstly, intraoperative frozen section was used selectively rather than routinely, generally in cases where results were anticipated to influence intraoperative decision-making; its limited and frequently non-diagnostic yield here likely reflects both sampling constraints and preoperative uncertainty, although prior literature suggests it may improve diagnostic accuracy and guide re-sampling. Secondly, the predominance of gliosis and normal parenchyma may further suggest sampling error. It is a possibility that the lesion was pathological, but the specific biopsy core attained was non-diagnostic (hence our TN vs. FN analysis). This could feasibly be driven by intralesional heterogeneity; this is particularly relevant in suspected gliomas where necrosis, edema, and viable tumor tissue coexist in the same lesions (see discussion below on intraoperative histology).

Furthermore, additional clinical decision-making factors also warrant clarification. In a small but relevant subset of cases (22%, *n* = 5/23) there was no clear overarching anticipatory diagnosis. In our retrospective chart review this was due to genuine diagnostic uncertainty, and biopsy was therefore pursued to resolve ambiguous but clinically/radiologically concerning findings, when and where it was considered safe to do so. Similarly, out of all of the pre-operative symptomatic categories, patients that were asymptomatic formed the largest sub-cohort (26%, *n* = 6/23). In these cases, the biopsy was undertaken to exclude potentially high-risk pathology based on concerning imaging findings, such as a possible neoplasm. In certain cases, such as these, biopsy indications are guided by diagnostic uncertainty and risk assessment following multi-disciplinary agreement rather than pure clinical symptomatology. Likewise, the relatively low (*n* = 2/23) number of patients that underwent subsequent neurosurgical intervention after the non-diagnostic biopsy. This can be appreciated when viewed through the lens of risk-management, given that many patients improved, remained asymptomatic, or indeed the fact that an alternative/new diagnostic clue came to light, pointing to a disease that was best addressed through medical management.

Additionally, it is important to comment on the evolution of the biopsy approach over the 15-year study period. As our experience with laser interstitial thermal therapy (LITT) continues to grow, many cases that may have been pure biopsies in the past have become biopsy followed by LITT when glioma or metastasis is suspected. In addition, the number of attending surgeons in our institution has grown as have our residency and neuro-oncology fellowship programs, meaning that as time goes on the breadth of experience has increased.

### Technological evolution

The technical execution of stereotactic biopsy plays a key role in diagnostic yield. At our institution, both robotic and conventional stereotactic platforms were employed. Among robot-assisted procedures, the non-diagnostic rate was 5.05%, compared to 6.88% across non-robotic biopsies (not significant). Trends in the literature suggest that robotic systems may enhance trajectory planning, entry-point precision, and procedural reproducibility [21, 22]. However, it is important to note that the timeline of ROSA use was clearly skewed between pre- and post-2019, limiting this conclusion; likewise, the experience of our surgical team evolves and improves over time, making a robot vs. non-robot comparison inherently tied to and biased to the impact of time.

Robotic systems such as ROSA, REMEBOT, and CAS-R-2 have introduced higher trajectory accuracy and improved reproducibility across operators. In a single-center series comparing ROSA and manual image-guided systems, the diagnostic yield was 97.4% for ROSA versus 93.3% for manual image-guided surgery (*p* < 0.05), with no significant difference in complication rates [23]. For REMEBOT, both large retrospective and prospective studies report diagnostic yields of 93–98%, with target point errors typically below 1.2 mm and low morbidity [24, 25]. A multicenter study comparing ROSA, REMEBOT, and CAS-R-2 found no significant difference in diagnostic yield among the three robotic systems (all approximately 94%), and no significant difference compared to manual techniques; lesion characteristics (enhancement, size, location) were more predictive of diagnostic success than the choice of robotic platform [26].

Further studies reveal the value of robotic platforms in minimizing target point error (TPE) and optimizing tangential entry angles, which are factors independently associated with diagnostic success [27, 28]. Robotic systems may also mitigate challenges associated with deep or small lesions. Reduced TPE was associated with higher diagnostic yield in a cohort of 584 patients undergoing robot-assisted biopsy [28, 29]. Alternative systems, including Stealth Autoguide (Medtronic, Minneapolis, USA) and Brainlab Varioguide (Brainlab, Munich, Germany), have also shown promising diagnostic yields with variable learning curves and operator-dependence, with diagnostic yields of 87.3% [30, 31]. Additionally, surgeon variability can account for 12% of diagnostic outcome differences [31].

Beyond navigation platforms, attention to biopsy technique and tissue handling can also improve yield. Needle selection, such as Laitinen versus Nashold types, can impact the quality and size of retrieved tissue cores. Differences in diagnostic material have been reported between these needle types, particularly in highly necrotic or hemorrhagic lesions [1]. Similarly, techniques such as the air-injection maneuver have also been used to confirm needle tract patency and potentially improve retrieval of diagnostically viable material [32, 33].

Emerging technologies in intraoperative diagnostics offer additional promise. One example is Stimulated Raman histology (SRH). SRH, in combination with deep convolutional neural networks (CNNs), allows for near real-time tissue classification intra-operatively. In a multicenter trial of 278 patients, CNN interpretation of SRH images achieved diagnostic accuracy comparable to standard pathology (94.6% vs. 93.9%). This approach may be particularly beneficial in cases where intraoperative pathology support is limited, or lesions are histologically subtle [34]. SRH can also aid in tumor subtype classification and molecular profiling (e.g., IDH, 1p/19q), with up to 93.9% accuracy for adult-type diffuse gliomas, suggesting a potential part in real-time personalized diagnostics [35].

### Re-biopsy outcomes and management of non-diagnostic biopsies

In our cohort, two patients underwent re-biopsy, with a subsequent diagnostic yield of 50% (although one was an open resection not a stereotactic biopsy, effectively lowering our re-biopsy diagnostic rate to 0%). Second biopsies can yield definitive diagnoses in 75%-90% of cases [7, 10, 36]. These findings reinforce the value of repeat tissue sampling when clinical suspicion remains high. However, re-biopsy is not always feasible due to lesion accessibility, patient frailty, or preference.

Strategies for managing these cases remain institution specific [36]. Some advocate for structured algorithms that include early multidisciplinary review, criteria for re-biopsy, and scheduled follow-up imaging. Others suggest that advanced imaging modalities, such as MR spectroscopy, diffusion tensor imaging, or PET, may help identify biopsy targets or predict non-diagnostic risk preoperatively [37]. In our cohort, 3 patients received PET, but this did not yield any further diagnostic certainty. In addition, taking biopsies from multiple locations of the same tumor (i.e. peripheral and central for example), is also an approach taken by some, although this varies significantly between and within institutions.

### Limitations

Our study has several limitations. The results were based on a relatively small number of non-diagnostic biopsy cases. In addition, we did not perform a comparison with patients who had diagnostic biopsy results; instead, we intentionally focused only on cases with non-diagnostic biopsies. Our intention was to investigate this small subset, rather than quantify the difference exactly between the diagnostic and non-diagnostic cohorts. Furthermore, there are, naturally, limitations to which point along the biopsy pathway the source of the non-diagnosis is, as the reason could be multi-fold: inappropriate patient selection at the outset with no biopsiable-tissue on pre-operative imaging; biopsy of non-diagnostic/indeterminate part of the lesion (e.g. hemorrhagic or mildly gliotic part of an otherwise clinically and radiologically evident lesion); or indeed the transfer, maintenance, staining and processing of tissue by the neuro-pathology team. Given the long and retrospective nature of this descriptive work, we were not able to query each surgeon for the reason they thought a patient might have had a non-diagnostic biopsy, as this would be both impractical and susceptible to significanat recall bias.

## Conclusions

Negative biopsy results (even if in the low single digit percentages) are almost inevitable and are reflected in the available literature. This reflects a complex spectrum of patient selection, neurosurgical center patient population, practice patterns of the wider multidisciplinary neuro-oncology teams, as well as technical expertise of the surgeon as well as neuropathologist. As evidenced by our TN vs. FN analysis, a non-diagnostic biopsy can also be the “correct” result. Improvements in intra-operative technology will always be welcome, although we postulate that the key question for a neuro-oncological service to be able to answer is what to do with the patients who have a negative biopsy.

## Data Availability

Data is available from the corresponding author.
